# Using of Radial-Shear Rolling to Improve the Structure and Radiation Resistance of Zirconium-Based Alloys

**DOI:** 10.3390/ma13194306

**Published:** 2020-09-27

**Authors:** Alexandr Arbuz, Anna Kawalek, Kirill Ozhmegov, Henryk Dyja, Evgeniy Panin, Anuar Lepsibayev, Sanzhar Sultanbekov, Rakhima Shamenova

**Affiliations:** 1 Collective Use Office, AEO Nazarbayev University, 53 Kabanbay Batyr Ave, Nur-Sultan 010000, Kazakhstan; rshamenova@nu.edu.kz; 2 Metal Forming Institute, Częstochowa University of Technology, ul. J.H. Dąbrowskiego 69, 42-201 Częstochowa, Poland; kawalek.anna@wip.pcz.pl (A.K.); kvozhmegov@wp.pl (K.O.); dyja@wip.pcz.pl (H.D.); 3 Metal Forming department, Karaganda Industrial University, Republic av. 30, Temirtau 101400, Kazakhstan; cooper802@mail.ru; 4 RSE National Nuclear Center of the Republic of Kazakhstan, Beybit atom st. 2B, Kurchatov 071100, Kazakhstan; A.Lepsibayev@mail.ru; 5 JSC Volkovgeologiya (NAC Kazatomprom), Bogenbay Batyr str. 168, Almaty 050012, Kazakhstan; sanzhar.stb@gmail.com

**Keywords:** UFG, severe plastic deformation, zirconium, radial-shear rolling, TEM, FEM-simulation, gradient microstructure

## Abstract

An overview of the prospects for the development of nuclear technologies and the conclusion of the relevant requirements for advanced structural materials, their classification and features were performed. In order to obtain a bar with a modified radiation-resistant outer layer, an experiment of radial-shear rolling under the most stringent conditions was carried out. For the same conditions, a FEM-simulation of sequential rolling in eight passes with a total compression of 70.7% (from a diameter of 37 mm to 20 mm) was conducted. For adequate simulation results a new material database for Zr-1%Nb alloy using plastometry investigations was generated. An experimental obtaining of a gradient-modified structure with an ultrafine-grained (UFG) periphery and an elongated rolling texture in the center of the bar was performed.

## 1. Introduction

The development of nuclear power is one of the main ways to provide people with energy in the long term in the future. The main concept of creating a new generation of nuclear reactors is to increase their safety level, increase their service life and power [1,2,3]. The development of nuclear technology is associated with a gradual increase in operating temperatures and pressures in the reactor core [2]. Also, the problem of creating small nuclear reactors with a particularly long autonomous operation, for stable energy supply in extreme conditions without maintenance [4], is relevant. At the same time, the main problem with increasing power, durability and safety is the degradation of structural materials under prolonged exposure to ionizing radiation in an aggressive environment and high temperatures.

This is especially true for the materials of the nuclear fuel cladding tubes and end plugs of the reactor fuel elements, because they separate the nuclear fuel from the reactor interior. The traditional material here is zirconium-based alloys [2,5]. The solution to this problem has far-reaching scientific and practical significance. This issue is also becoming more relevant due to increasing requirements for the safety of NPS operation.

One of the methods to increase the level of mechanical properties and radiation resistance is the use of ultrafine-grained (UFG) and nanostructured materials (NS) [6]. Structural elements made of Zr-based alloys have a grain size of about 5–20 microns. The UFG state, with a grain size of less than 1000 nm, significantly changes the mechanical and physical properties of the material. The strength increases several times while maintaining a sufficiently high level of plasticity, which is very beneficial for structural materials. These materials, their properties and features of production are described in detail in the works of R.Z. Valiev, T. Langdon, and others [6,7,8,9].

Such materials, due to their small grain size, contain a large number of grain boundaries in the structure, which play a decisive role in the formation of their physical and mechanical properties. The main goal of works on grinding the structure of metals and alloys is to obtain equiaxed structures with large angles of crystallographic disorientation and grain size less than 1 µm, close to those shown in [6].

The physical mechanism for increasing radiation resistance also consists in the fact that UFG and nanostructured metals have a much larger number of grains per volume unit, and therefore a relatively large proportion of intergranular boundaries in the volume of the body. In the case of a radiation defect, due to the proximity of grain boundaries, the defect quickly reaches the grain boundaries that serve as the surface of the drain and disappears. In particular, a decrease in the proportion of amorphization, radiation hardening and swelling was recorded for nanostructured alloys obtained by equal-channel angular pressing [10,11] the most common method for obtaining UFG structures. The study of radiation resistance of this class of materials is most actively conducted in the nuclear centers of the United States [12], France [13], Japan [14] and other countries [15,16].

The production of ultrafine-grained and nanostructured state is possible either by sintering ultradispersed powders, or by the method of severe plastic deformation (SPD). The use of SPD eliminates the presence of residual porosity characteristic of sintering powders, but has more stringent requirements for the process conditions:

(1) achieving high degrees of strain for grain grinding (ε > 6–8);
(2) formation of high hydrostatic pressure that prevents sample destruction and annihilation of crystal lattice defects (1 GPA or higher);
(3) deformation at temperatures about 0.3 of the melting point and below to exclude the phase transition α→α + β in a Zr-based alloy [17];
(4) providing turbulence and non-monotonous deformations that contribute to the formation of high-angle intergrained boundaries.

The most popular methods that implement these conditions are equal channel angular pressing (ECAP) in 6–8 cycles or more [11] and high-pressure torsion (HPT), as the evolution of Bridgman anvil [8]. In addition, SPD methods based on rolling have been developed [18,19].

These methods make it possible to obtain ultrafine grain for a number of materials (there are many times confirmed protocols for various materials) and, judging by the ratio of publications, they are most in demand for scientific research. These methods are the main ones in more than 90% of publications on UFG materials and in all publications on radiation resistance research. However, when convenient for laboratory use, both methods have two major drawbacks: a limitation in the size of the samples obtained and extremely low production technology, which exclude their industrial use. These disadvantages can be eliminated by using a method of intense deformation, such as radial-shear rolling, which also allows to obtain a UFG state, but already in long round bars with some features of the structure distribution [20,21,22]. The development of this method will become advantageous for the industrial application of products made of radiation-resistant UFG materials.

**Figure 1:**
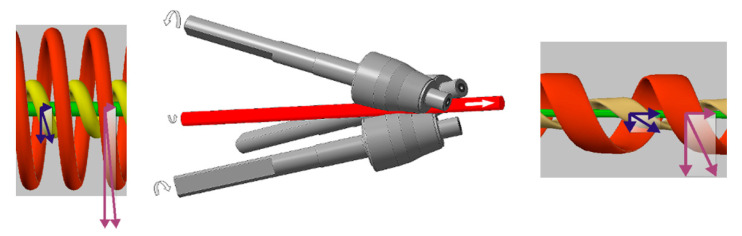
Scheme of radial-shear rolling and features of the metal flow of the axial, intermediate and peripheral zones of the workpiece.

During radial-shear rolling, in the deformation zone a stress state scheme close to all-round compression with large shear deformations and high metal flow turbulence is implemented. The scheme of radial-shear rolling and features of metal flow is shown in Figure 1.

The main features of radial-shear rolling include non-monotonicity and turbulence of deformation, as well as differences in the plastic flow and structure of different zones of the workpiece due to the trajectory-speed features of the process [21]. The difference between the process and the well-known screw (or helical) rolling used for sewing pipes is that a three-roll scheme and special angles of convergence of the rolls are used, which provide comprehensive compression along the cross-section of the sample in contrast to stretching in traditional screw rolling. The angle of inclination of the roll surface trajectories was 18°, while the angle of inclination of the roll generating line to the neutral axis of the rolling process in the deformation part was equal to 9°. Because of to these metal flow features, the most intense shear deformations are localized in the zone of intersection of the metal sliding lines—the circular cross-section zone typical for the three-roll scheme. In the outer layer, each small trajectory-oriented element is subjected to compression deformation along the radius of the workpiece, compression deformation in the flow direction (along the helical trajectory) and stretching deformation across the helical trajectory. At the same time, it is important that there is a constant gradient of velocities and flow directions along the radius, which also adds additional shear elements to the overall complex picture of the stress-strain state. Structural elements of a metal subjected to an expanding flow with a two-way precipitation (along the trajectory and along the radius) take the form of isotropic isolated particles of high dispersion [21].

The speed of particles in the axial fiber and its length, as well as in longitudinal rolling, increases in proportion to the extraction coefficient. The cross section of the central flow tubes decreases. The processing of the metal structure is based on the type of longitudinal rolling in calibers with multi-sided compression or pressing. Elements of the structure are stretched and refined to form a characteristic structural banding. These features are described and illustrated in detail in the works of S.P. Galkin [21].

This method largely implements the necessary conditions described above for the formation of the UFG structure. At the same time, the best conditions (and presumably—the structure and properties) are obtained in the peripheral part of the circular cross—section of the sample for its entire length, which is acceptable from the point of view that the intended final product is fuel rod tubes. The length of the bar is limited only by the technological parameters of a particular stand. These two facts cause a high interest in the study of the influence of this method on changes in the structure and properties of the zirconium alloy and testing of radiation resistance of the resulting structure in the future. In addition, studies aimed at obtaining the UFG structure of zirconium alloys by radial-shear rolling have not yet been conducted, which also determines the scientific novelty of the work.

The main purpose of this work is to evaluate the applicability of the radial-shear rolling method for grinding the structure of a zirconium alloy to a level that theoretically provides an increase in its radiation resistance. The goal is achieved by solving the following tasks:

(1) Plastometric study of the rheological properties of the selected zirconium alloy in order to obtain initial data on the regularities of the material behavior in the conditions of radial-shear rolling and its detailed study by computer modeling;
(2) Computer simulation of the radial-shear rolling process in order to assess the compliance of the stress-strain state scheme implemented by the mill with respect to the above-mentioned parameters optimal for the formation of the UFG structure, preparation of the experiment and interpretation of its results;
(3) Experimental rolling at the maximum mechanical and technological modes of the mill in order to assess the maximum possible level of influence of the radial-shear rolling process on the change in the structure of the zirconium alloy;
(4) Study of the resulting structure and answer to the main question of the study on the applicability of radial-shear rolling for grinding the structure of a zirconium alloy to a level that theoretically provides an increase in its radiation resistance.

In the future, based on the rheological and experimental data obtained here, an extended theoretical and experimental study of the regularities of changes in the structure of the zirconium alloy will be carried out, as well as irradiation and experimental study of the radiation resistance of the material obtained here.

## 2. Materials and Methods

One of the most common zirconium-based alloys is the Zr-1%Nb alloy. This alloy is used as a material for shells (nuclear fuel cladding tubes) and end plugs, as well as spacer grids of fuel elements of thermal neutron reactors (PWR, WWER).

To understand the metal flow in relation to the temperature-speed conditions of radial-shear rolling of the Zr-1%Nb alloy, taking into account the thermal effect of plastic deformation, plastometric tests were performed. Plastometric tests were carried by uniaxial compression of cylindrical samples with a working part diameter of 10 mm in the range of strain rates of 0.5–15 s^−1^ and a temperature of 20–650 °C. The tests were performed under continuous loading conditions on the “Gleeble 3800” plastometric unit using the “Pocket Jaw” module. The “Gleeble 3800” plastometric unit allows to simulate the conditions of various metal forming processes and perform tests with high measurement accuracy. The temperature fluctuation is no more than ±1.0 °C. The measurement error of the loading force is ±1.0 kg per 1 ton. The accuracy of movement of the working traverse is not more than ±0.01 mm. Other parameters are derived from the specified values. The test temperature was controlled using a chromel-copel thermocouple welded to the central part of the sample on the Thermocouple welder attached to the Gleeble 3800 set. Thin graphite-based gaskets were used as a lubricant during the tests. The ISO-T working strikers were additionally lubricated with OKS 255 graphite grease after each test.

The Deform software (SFTC, Columbus, Ohio, USA) was chosen for computer simulation, which allows simulating the metal forming process of any complexity. Modeling is performed by the finite element method (FEM). To create a model of radial-shear rolling, it was decided to use the parameters of the existing SVP-08 mill installed at Częstochowa University of Technology. The initial billet with a diameter of 37 mm and a length of 150 mm was rolled on a mill with the compressions specified in Table 1. Compressions were determined based on previous experience of using this mill with other materials [20,21,22]. The material of the billet is Zr-1%Nb alloy. Since this material is not available in the Deform database, the results of plastometric studies were imported. In this way, a new library of research material for the Deform was created.

The heating temperature 530 °C for rolling was chosen, as the maximum possible to avoid a phase transition of the alloy to the region of brittle β-Zr [17,21]; the rotation speed of the rolls was equal to 100 rpm, as the nominal value on the SVP-08 mill. The friction coefficient at the contact of the billet and rolls was assumed to 0.7, as the recommended value for hot rolling in Deform. To create the roll geometry, Kompas-3D v. 18 software was used by saving the finished geometry in the STL format. When rolling, the rolls were taken by rigid bodies, and the material of the billet was elastic-plastic.

**Figure 2:**
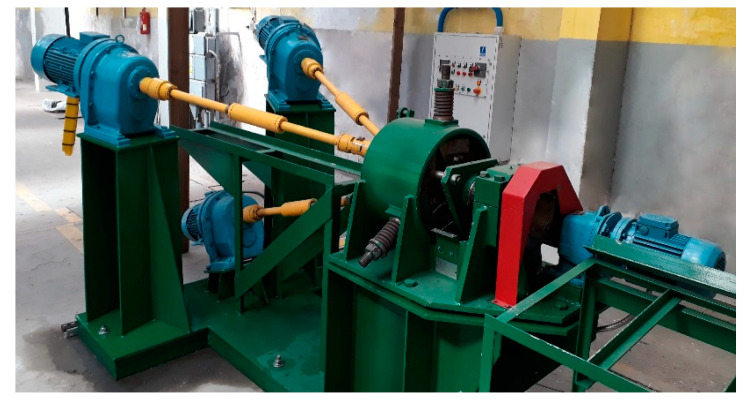
SVP-08 radial-shear rolling mill.

Verification of computer simulation results was carried out at the SVP-08 mill (ZAO “ISTOK ML”, Moscow, Russia) at the Częstochowa University of Technology. The mill was designed at NUST MISIS. The appearance of the mill is shown in Figure 2.

The SVP-08 mill is designed for hot deformation of solid round bars made of almost any metal materials, including low-plastic, continuous cast and powder. Rolling of bars with a diameter from 40 mm to 8 mm is carried out in a three-roll cage of a special rigid structure by compressing the blanks along the diameter in one or several passes using rolls with a diameter of 90 mm of special calibration, and if necessary, with intermediate preheats.

This mill was chosen for conducting experiments to study the effect of radial-shear rolling on the microstructure of zirconium, because the mill is characterized by a wide range and high rigidity of the cage and ease of operation. Previously, using a similar type of mill, an ultrafine structure with equiaxed grains of 300–700 nm was obtained on samples made of AISI-321 stainless austenitic steel, which is also widely used in nuclear power engineering [22,23,24].

A rod of Zr-1%Nb alloy with an initial diameter of 40 mm obtained by hot pressing with an extract of µ ≈ 25 at a temperature of 650 °C was used for rolling. Then the rod was mechanically processed to a diameter of 37 mm and then was heated and rolled.

**Figure 3:**
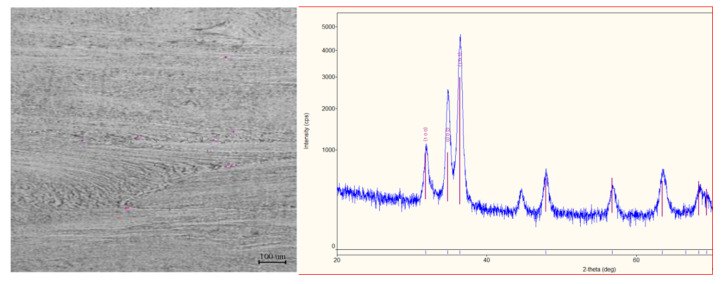
Microstructure of longitudinal section (×200) and diffractogram of the initial Zr-1% Nb alloy.

The mechanical properties of the initial pressed bar were as follows: tensile strength = 470 MPa, yield strength = 348 MPa, elongation = 34%. The phase composition of the initial bar was studied on the DRON-6 diffractometer (RPE “Thunderbird”, Saint Petersburg, Russia). The phase composition of the initial bar is two-phase. The matrix consists of a more plastic α-Zr (HCP, lattice parameter a = 0.3231 nm) and contains less than 1% β-Zr particles with a BCC lattice (a = 0.361 nm) with 17% Nb dissolved in it. The initial structure of the periphery and central region is shown in Figure 3. Bars after hot pressing have a deformed fibrous structure along the length.

The heating temperature was set to 530 °C, and the initial bar with a diameter of 37 mm was heated in a preheated muffle furnace for 40 min. An infrared thermal imaging camera ThermaCAM SC640 Thermovision Thermometer (FLIR Systems, Nashua, New Hampshire, USA) aimed at the deformation zone was used to control the temperature mode.

The phase composition was studied using the XRD method on the automated high-resolution θ-θ multi-purpose X-ray diffractometer (XRD) SmartLab (Rigaku Corp., Tokyo, Japan) with expert system Guidance software, 1.5 kW sealed X-ray tube, CBO optics, D/teX Ultra 250 silicon strip-detector (Rigaku Corp., Tokyo, Japan).

The initial microstructure was studied using optical microscopy on an etched sample at X200 magnification in a light field using an OLYMPUS GX-51 microscope (Olympus Co., Tokyo, Japan). Grinding and polishing was carried out on the Sapphire-520 machine (ATM Gmbh, Mammelzen, Germany)

The deformed microstructure was studied by transmission electron microscopy (TEM) in a light field and by electron diffraction on a JEM-1400 Plus microscope (Jeol Ltd., Tokyo, Japan) at an accelerating voltage of 80 kV and x magnification.

Sample preparation for TEM was carried out by the following method. A 10 mm long sample was cut from the central part. Then it was cut in half and several consecutive longitudinal thin (0.3 mm) sections of the axial area were taken. Thus, the most informative longitudinal section for radial shear rolling is used for microstructure analysis. Some of the samples were used for testing sample preparation modes. The cutting was carried out on a high-precision cutting machine Brilliant-220 (ATM Gmbh, Mammelzen, Germany) with a water-cooled abrasive wheel at a speed of 500 rpm and a feed of 15 microns/s.

Then, using the electrolytic jet thinning method, the final TEST samples were made in 2 stages at the TenuPol-5 plant (Struers, København, Denmark) in A3 electrolyte (600 mL Methanol, 360 mL Butylcellosolve, 60 mL Perchloric Acid). At the first stage, preforms of TEM objects with a diameter of 3 mm were etched from a solid plate with a thickness of 0.3 mm; at the second stage, these preforms were thinned to form a hole at a voltage of 20 V.

## 3. Results and Discussion

### 3.1. Rheological Properties of Zr-1%Nb Alloy

**Figure 4:**
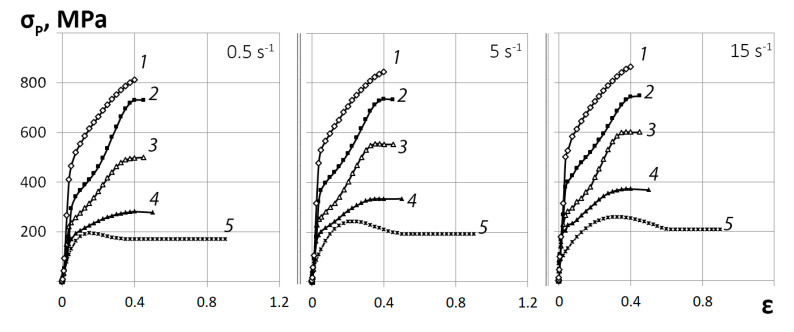
Flow curves of Zr-1%Nb alloy obtained using the “Gleeble 3800” plastometer by compression method in the range of strain rates 0.5–15 s^−1^ at temperatures: 1–20 °C; 2–200 °C; 3–350 °C; 4–500 °C; 5–650 °C.

According to the plastometric results, the graphs of the “stress-strain” flow curves for the Zr-1%Nb alloy obtained in the range of strain rates of 0.5–15 s^−1^ and temperature of 20–650 °C were constructed (Figure 4).

It is shown that with an increase in temperature from 20 to 650 °C, the deformation resistance decreases by about ~77%. An increase in the strain rate from 0.5 to 15 s^−1^ leads to an increase in the deformation resistance. Thus, at a temperature of 20 °C, the increase in the deformation resistance is no more than 8%. At 650 °C the increase in deformation resistance is approximately ~25%. This difference in the influence of the strain rate can be explained by the thermal effect of plastic deformation. At temperatures close to room temperature, the thermal effect is stronger than at higher temperatures.

As the temperature and strain rate increase, the nature of the flow curves changes. In the temperature range of 350–500 °C, there is a noticeable decrease in the coefficient of hardening in the deformation range of 0.3–0.4 on the flow curves. This can be explained by the increasing number of sliding systems involved in deformation and the ongoing processes of dynamic return. At a temperature of 650 °C, the flow curves are domed with a maximum deformation resistance of 0.15 to 0.30 depending on the strain rate. Then the increase in deformation resistance becomes zero, and the flow curves go to a steady stage. This phenomenon is typical for metals and alloys of the hexagonal close-packed (HCP) system. These materials are characterized not only by a significant thermal effect in conditions of cold and warm deformation with high speeds, but also by a pronounced anisotropy of properties with the appearance of textural heterogeneity.

### 3.2. Computer Simulation of Radial-Shear Rolling of Zr-1%Nb Alloy

The results of plastometric studies were used as input parameters for computer modeling. Detailed information can be found in “Supplementary Materials” section. To study the level of metal processing during deformation using FEM-simulation, the parameter “equivalent strain” is usually considered. In many sources, it is often indicated as “accumulated strain” because it is a cumulative parameter, i.e., after the load is removed, this parameter is not reset, unlike the stress.

**Figure 5:**
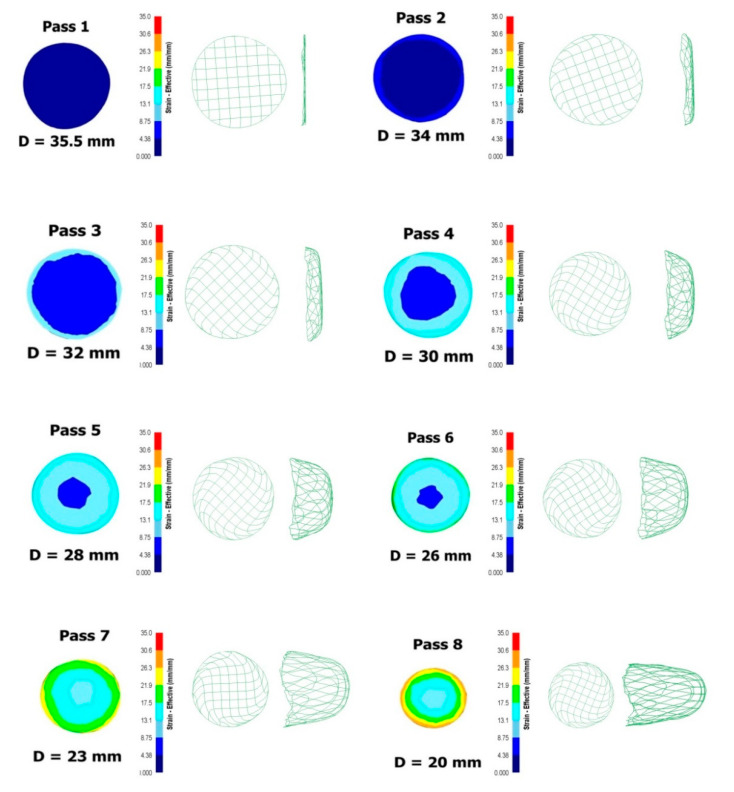
Equivalent strain and fixed grid in the cross section of a zirconium bar after eight passes of radial-shear rolling.

Since radial-shear rolling is a cross type of rolling, it is advisable to study the equivalent strain not only in the longitudinal, but also in the cross-section of the workpiece—this will allow to evaluate not only the numerical values of the parameter, but also the nature of its distribution over the cross-section during deformation. When analyzing the equivalent strain (Figure 5), it was found that the distribution of this parameter fully corresponds to the cross type of deformation when the workpiece rotates around its axis, since in the cross section, clearly visible ring zones of strain development. At the same time, relatively large unevenness of the strain distribution in the radial direction can be noted.

In the axial zone (0–35% of the radius from the center), the strain level after eight passes is about 9.5. In the peripheral zone (35–80% of the radius from the center), the intensity of shear deformation increases, here the level of strain is 19.5–21.5. In the surface zone (80–100% of the radius from the center), the maximum effect of shear deformation is observed, here the strain level is 29–30.6.

Also, a fixed FlowNet grid was constructed in the cross section of the workpiece, which, unlike the finite element mesh, is never rebuilt, but only deformed according to the nature of the metal flow. Considering this grid in the transverse direction, it can be noted that the curvature of the cells occurs only in the surface layer of the workpiece, which indicates the turbulent nature of the metal flow. The cells in the central zone practically do not change their shape, this effect indicates the laminar flow of the metal. In the longitudinal direction of the grid with increasing number of passes gets a funnel shape, and the intensity of curvature depends on the magnitude of compression—in the last passes, where the amount of compression is 3 mm, the intensity extraction is most noticeable.

**Figure 6:**
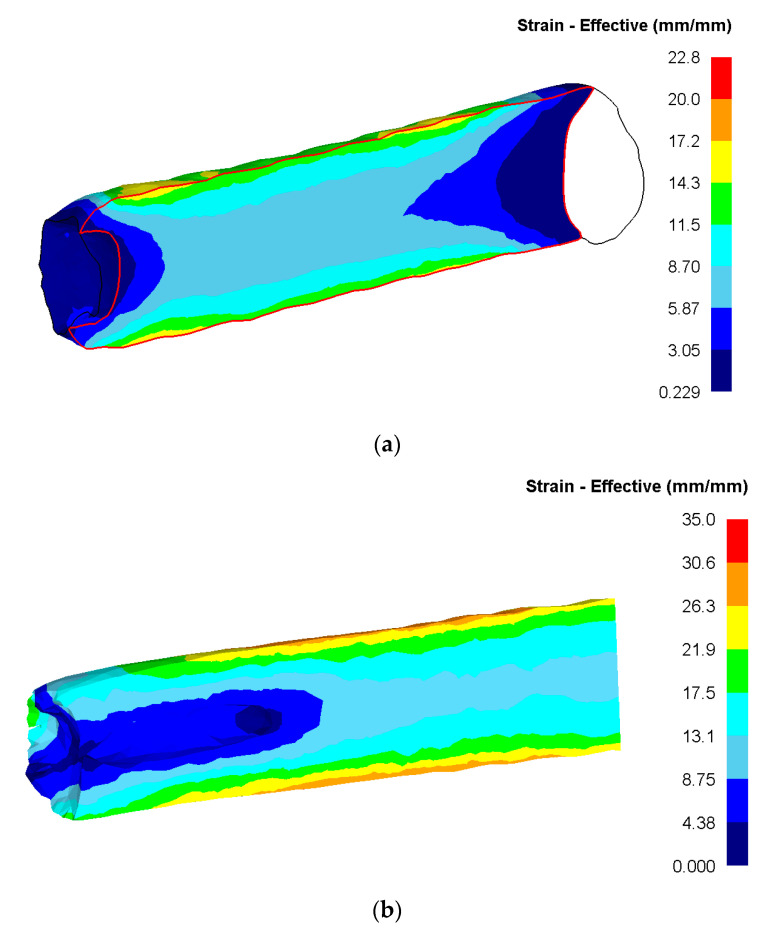
Equivalent strain in the longitudinal section of the bar: (**a**)—rolling for a diameter of 30 mm; (**b**)—rolling for a diameter of 20 mm.

When considering the longitudinal section of the bar (Figure 6), the uneven distribution of this parameter in cross direction is clearly visible. With the least developed axial zone, the maximum level of deformation develops in the surface layers. At the same time, the character of the strain distribution in the longitudinal direction is fairly uniform.

**Figure 7:**
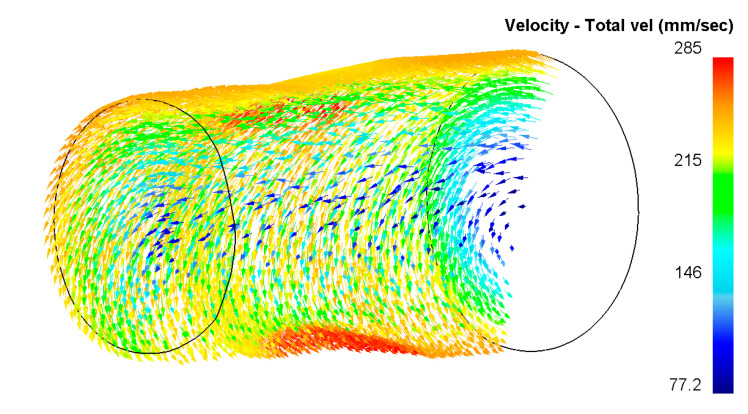
Character of metal flow during radial-shear rolling.

Such character of strain distribution can be easily explained using the image of the vectors of the metal flow directions (Figure 7). From the longitudinal section of a billet is clearly seen that in the surface layers of the workpiece by radial-shear rolling of the formed vortex nature of the metal flow conducive to modifications to microstructure, to give it a more equiaxed shape and crushing it to a UFG size. At the same time, in the axial zone, the character of the metal flow is laminar, where the vortex direction is practically not formed and the metal flows only in the longitudinal direction.

**Figure 8:**
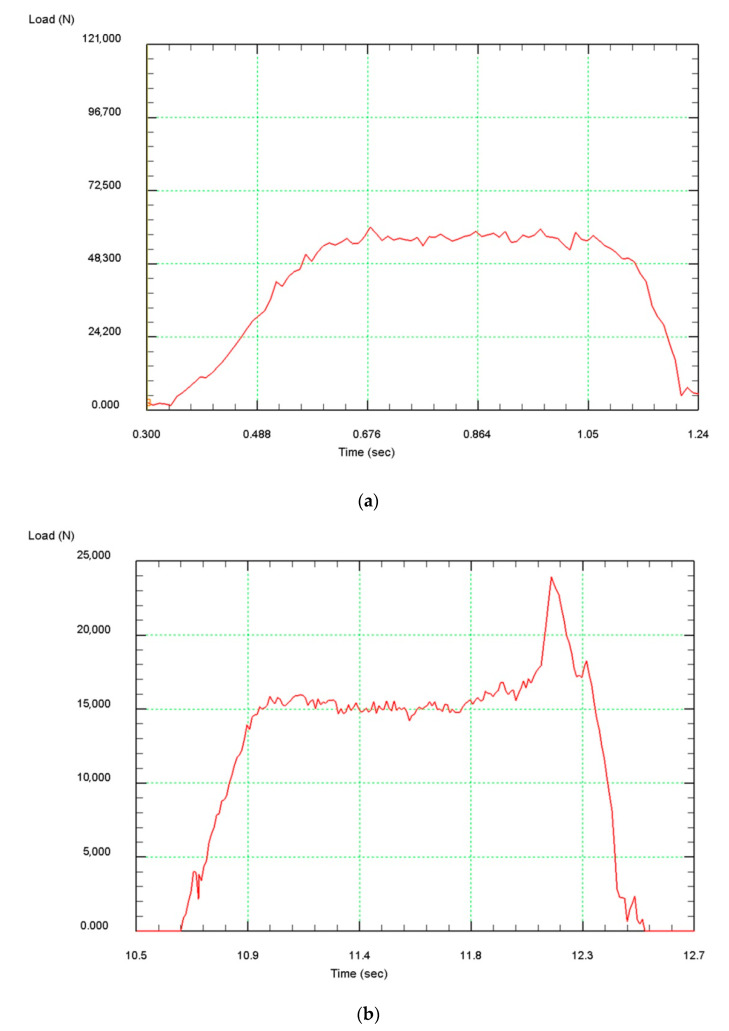
Character of force graphs for radial-shear rolling: (**a**)—total compression up to 20% (1st pass); (**b**)—total compression over 20% (8th pass).

The analysis of the metal force on the rolls during rolling at the SVP-08 mill of the Zr-1% Nb alloy blanks was also performed. Figure 8a shows a typical force graph for radial-shear rolling. Gradually increasing during the capture of the billet by rolls, the force graph becomes constant when the rolling process is established. When the metal leaves rolls, the force is reducing gradually to zero due to the appearance of rear shrinkage cavity. A different type of force distribution is obtained after achieving a total compression of 20% (Figure 8b). Despite the fact that the overall level of force decreases with the growth of the total compression, a sharp jump in force is clearly visible when the billet exits the rolls.

**Figure 9:**
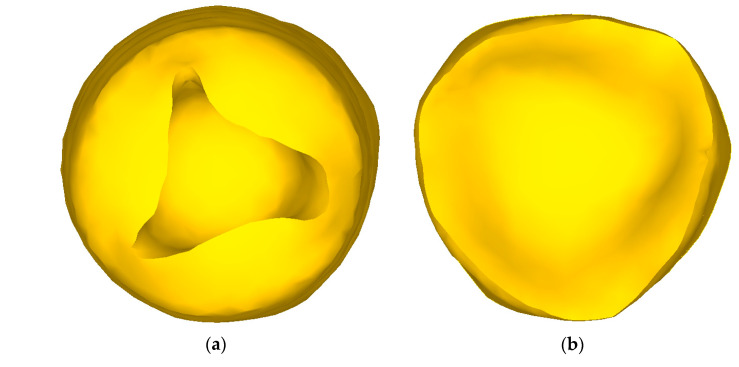
Shape of the billet ends in radial-shear rolling with a total compression of more than 20%: (**a**)—front end; (**b**)—back end.

This phenomenon can be explained at consideration the shape of the ends of the workpiece (Figure 9). At both ends of the workpiece due to the transverse type of deformation, shrinkage cavities (funnel–shaped blind holes) are formed, which are the result of a significant misalignment of the metal flow rates in the radial direction. With a small total compression of up to 20%, the shape of the shrinkage cavity remains round. As the total compression increases, the shrinkage cavity at the front end of the billet (Figure 9a) becomes triangular (in some cases—square), while the external shape of the billet remains always round due to the harsh capture conditions by rolls.

A shrinkage cavity is also formed at the back end, but the nature of its formation is different (Figure 9b). Due to the fact that the front shrinkage cavity is formed during capture, and the rear shrinkage cavity is formed during steady rolling, its depth is much less here. At the same time, because the edges of both ends of the billet are compressed by different sections of the roll, the back end of the billet acquires an irregular shape, close to a triangular one. The length of this area depends on the depth of the back shrinkage cavity. During rolling of this area, there is a jump in force due to a sharp increase in absolute compression.

In general, it was noted that the radial-shear rolling of a zirconium alloy bar in all eight passes creates a force that does not exceed the permissible value of 100 kN. Table 2 shows the values of the forces per passes.

Based on the results of computer modeling, recommendations for rolling bars of Zr-1% Nb alloy to ensure high processing of the structure without breaking the continuity of the metal were developed.

### 3.3. Radial-Shear Rolling and Microstructure Changes of Zr-1%Nb Alloy

Rolling was carried out at the maximum mechanical and technological conditions of the mill, and there were several cases of jamming of the rolls. After the rolling pass the rod was quickly removed and placed in the furnace for heating to the initial temperature, which was recorded by a thermal imager, and the rolling was continued in steps of 1.5–3 mm until the diameter of 20 mm was reached. This diameter is the technological limit for large rolls installed in the mill. Rolling to smaller diameters requires transshipment and replacement of rolls and cassettes, which takes time. Therefore, rolling to smaller diameters from a single heating is not possible.

**Figure 10:**
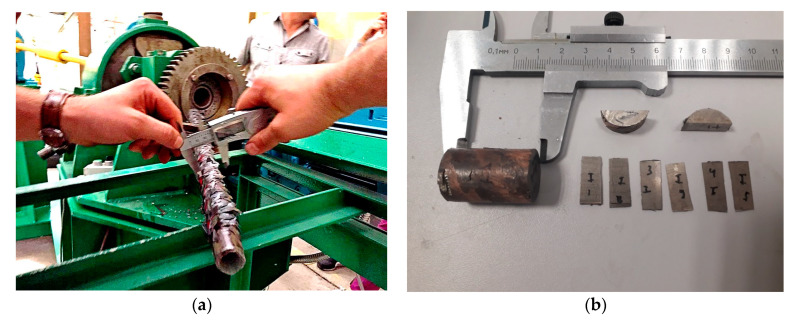
Workpiece after radial-shear rolling (**a**) and samples from the final bar (**b**) to study the microstructure.

**Figure 11:**
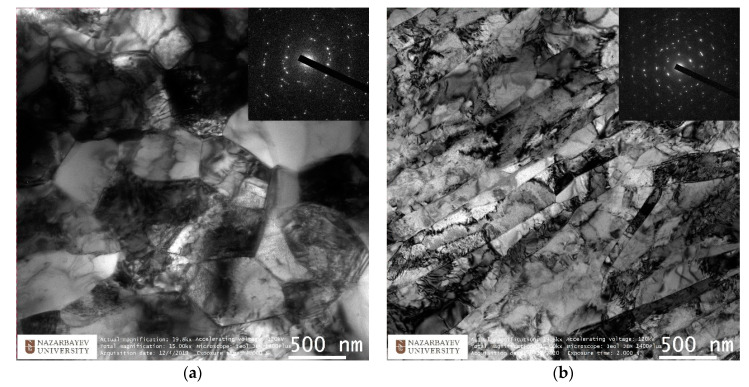
Structure of Zr-1%Nb alloy after radial-shear rolling (x): (**a**)—periphery, (**b**)—center.

A short-term increase in temperature from 50 to 150 °C was caused by the thermal effect of plastic deformation, which, with small absolute compressions, indicates the occurrence of intense shear deformations. The rolled rod was cooled in the air, from the central part the samples were taken in the form of two segments (10 mm and 30 mm long) to study the microstructure changes. Experimental rolling and selected samples are shown in Figure 10. The resulting TEM-images of the thin structure of the longitudinal section in the peripheral and axial regions are shown in Figure 11.

The electron images represent equiaxed dislocated fine grains with sizes from 500 to 1000 nm. Electron diffraction obtained from these regions also confirmed the absence of oriented texture and showed the presence of high-angle intergrain boundaries. This type of structure is the most optimal for achieving high properties and is suitable for further SPD processing in the nanostructured region.

The structure of the central region has also changed. Instead of large grains with no apparent orientation in the center, after radial-shear rolling, a mixture of long narrow elongated strongly deformed grains typical of strong uniaxial deformation (width from 100 nm and length of several 5–10 or more microns) and individual large grains with a size close to the original grains of 5–10 microns was formed. Sharp knife borders between the grains are clearly visible, which differs from the previously existing fibrous structure. At the same time, the rolling direction is clearly visible, and the preferred orientation of the grains corresponds to it, as indicated by slightly elongated fused diffraction reflexes on the electronogram. The obtained data show a significant transformation of the original fibrous structure both at the periphery and in the center of the sample. The approximate location of the study zones on the TEM sample corresponds to 2 mm from the edge of the sample for image of the peripheral zone and the center of the sample axis for image of the center. Thus, it can be argued that UFG structure is obtained to a depth of at least 2 mm with a total diameter of 20 mm. This work shows the principal possibility of obtaining a structure suitable for the description of radiation-resistant UFG structure using radial-shear rolling method.

## 4. Conclusions

This approach to determining rolling modes has shown high material and time efficiency. Based on the results of computer modeling, verification was carried out, which showed high convergence of the results of modeling and laboratory experiment. We can draw a conclusion about the applicability of radial-shear rolling in the deformation of bars made of a zirconium-based alloy Zr-1%Nb to obtain a UFG structure. It is advisable to consider the application of the radial-shear rolling process mainly towards the end of the production cycle of products.

Computer simulation of the radial-shear rolling process of Zr-1%Nb alloy bars was performed. For adequate simulation results, a new database of this material was created based on the results of plastometric tests by uniaxial compression of cylindrical samples in the range of strain rates of 0.5–15 s^−1^ and a temperature of 20–650 °C.

In the process of radial-shear rolling of bars along the route 37 mm→20 mm with a total deformation of ε ≈ 70%, an equiaxed ultrafine-grained microstructure was obtained. This microstructure was formed at the periphery of the sample at least 2 mm in depth, judging by the position of the analysis area of the peripheral TEM sample. In the center of the sample, a predominantly oriented rolling texture was formed with an admixture of larger grains of 5–10 microns. It is important to note that the grains of the outer UFG zone are equiaxed with predominant high-angle boundaries and large crystallographic disorientation of the lattice. Such a structure should provide an increase in operational properties, in particular, radiation resistance [12,13,14,15]. The resulting microstructure is in good agreement with the research data [6,7,18,19,20,23,24].

Mini radial-shear rolling mills can be used in research work on zirconium alloys as an effective means of warm plastic deformation (at 0.2–0.4 of melting temperature). In this case, the value of one-pass compression is recommended to assign no more than 15% when rolling bars from the Zr-1%Nb alloy at the SVP-08 mill.

Obtained structure in the course of this work will be further studied at the DC-60 accelerator (Institute of Nuclear Physics, Nur-Sultan, Kazakhstan) to conduct a comparative analysis of the behavior of the metal with UFG and traditional structures. Rolled bars with diameters of 20 mm will be used as semi-finished products for the production of nanostructured zirconium in long bars with a diameter of 14–15 mm. 

## Figures and Tables

**Table 1:** Rolling parameters of zirconium bar with diameter of 37 mm on the SVP-08 mill.

Number of Pass	D0, mm	D1, mm	Compression, mm
1	37	35.5	1.5
2	35.5	34	1.5
3	34	32	2
4	32	30	2
5	30	28	2
6	28	26	2
7	26	23	3
8	23	20	3

**Table 2:** Rolling forces per passes.

Number of Pass	One-Pass Compression, mm	Total Compression, mm (%)	Stable Force, kN	Force on the Jump, kN
1	1.5	1.5 (7.94)	57.8	-
2	1.5	3 (15.5)	50.4	-
3	2	5 (25.2)	45.2	52.3
4	2	7 (34.2)	39.4	47.6
5	2	9 (42.7)	32.3	41.7
6	2	11 (50.6)	27.7	39.6
7	3	14 (61.3)	20.4	31.4
8	3	17 (70.7)	15.2	24.5

## Supplementary Materials

The Deform material database and plastometry results for Zr-1%Nb alloy for in the range of temperatures 20–650 °C and strain rates 0.5–15 s^−1^ are available online at http://dx.doi.org/10.17632/pg9wfwdxmz.1.

## References

[1] Zinkle S.J., Busby J.T. (2009). Structural materials for fission & fusion energy. Mater. Today.

[2] Mansur L.K., Rowcliffe A.F., Nanstad R.K., Zinkle S.J., Corwin W.R., Stoller R.E. (2004). Materials needs for fusion, Generation IV fission reactors and spallation neutron sources—Similarities and differences. J. Nucl. Mater..

[3] Zinkle S.J., Was G.S. (2013). Materials challenges in nuclear energy. Acta Mater..

[4] Ingersoll D.T., Colbert C., Houghton Z., Snuggerud R., Gaston J.W., Empey M. Can Nuclear Power and Renewables be Friends?. Proceedings of the ICAPP 2015.

[5] Yu H., Dong Q., Yao Z., Zhang H.K., Kirk M.A., Daymond M.R. (2019). In-situ study of heavy ion irradiation induced lattice defects and phase instability in β-Zr of a Zr–Nb alloy. J. Nucl. Mater..

[6] Valiev R.Z., Islamgaliev R.K., Alexandrov I.V. (2000). Bulk nanostructured materials from severe plastic deformation. Prog. Mater. Sci..

[7] Langdon T.G. (2006). The characteristics of grain refinement in materials processed by severe plastic deformation. Rev. Adv. Mater. Sci..

[8] Xu C., Horita Z., Langdon T.G. (2007). The evolution of homogeneity in processing by high-pressure torsion. Acta Mater..

[9] Tsay K., Arbuz A., Gusseynov N., Nemkaeva R., Ospanov N., Krupenkin I. (2016). Refinement of the microstructure of steel by cross rolling. J. Chem. Technol. Metall..

[10] Shamardin V.K., Goncharenko Y.D., Bulanova T.M., Karsakov A.A., Alexandrov I.V., Abramova M.M., Karavaeva M.V. (2012). Effect of neutron irradiation on microstructure and properties of austenitic AISI-321 steel, subjected to equal-channel angular pressing. Rev. Adv. Mater. Sci..

[11] Segal V.M. (2004). Engineering and commercialization of equal channel angular extrusion (ECAE). Mater. Sci. Eng. A.

[12] Nita N., Schaeublin R., Victoria M. (2004). Impact of irradiation on the microstructure of nanocrystalline materials. J. Nucl. Mater..

[13] Etienne A., Radiguet B., Cunningham N.J., Odette G.R., Valiev R., Pareige P. (2011). Comparison of radiation-induced segregation in ultrafine-grained and conventional 316 austenitic stainless steels. Ultramicroscopy.

[14] Matsuoka H., Yamasaki T., Zheng Y.J., Mitamura T., Terasawa M., Fukami T. (2007). Microstructure and mechanical properties of neutron-irradiated ultra-fine-grained SUS316L stainless steels and Ni–W alloys. Mater. Sci. Eng. A.

[15] Wurstera S., Pippana R. (2009). Nanostructured metals under irradiation. Scr. Mater..

[16] Maksimkin O.P., Gusev M.N., Tsai K.V., Yarovchuk A.V., Rybalchenko O.V., Enikeev N.A., Valiev R.Z., Dobatkin S.V. (2015). Effect of Neutron Irradiation on the Microstructure and the Mechanical and Corrosion Properties of the Ultrafine Grained Stainless Cr-Ni Steel. Phys. Met. Metallogr..

[17] Dyja H., Kawałek A., Ozhmegov K. (2019). Experimental studies on Zr–1%Nb alloy properties in technological conditions of cold pilger tube rolling process. Arch. Civ. Mech. Eng..

[18] Kawalek A., Dyja H., Markowski J. (2003). Effect of asymmetrical rolling on broadening of the product line of rolled sheets. Metalurgija.

[19] Knapiński M., Dyja H., Kawałek A., Kwapisz M., Koczurkiewicz B. (2013). Physical simulations of the controlled rolling process of plate X100 with accelerated cooling. Solid State Phenomena..

[20] Lopatin N.V., Salishchev G.A., Galkin S.P. (2011). Mathematical modeling of radial-shear rolling of the VT6 titanium alloy under conditions of formation of a globular structure. Russ. J. Non-Ferr. Met..

[21] Galkin S.P. (2014). Radial shear rolling as an optimal technology for lean production. Steel Transl..

[22] Naizabekov A.B., Lezhnev S.N., Dyja H., Bajor T., Tsay K., Arbuz A., Gusseynov N., Nemkaeva R. (2017). The effect of cross rolling on the microstructure of ferrous and non-ferrous metals and alloys. Metalurgija.

[23] Naizabekov A., Lezhnev S., Arbuz A., Volokitina I., Panin E. (2019). The development and testing of a new method of qualitative analysis of the microstructure quality, for example of steel AISI 321 subjected to radial shear rolling. Phys. Scr..

[24] Naizabekov A., Lezhnev S., Arbuz A., Panin E. (2018). Obtaining of long-length rods with ultrafine-grained structure by the radial-shear rolling. IOP Conf. Ser. Mater. Sci. Eng..

